# Transcriptome Analysis of Bread Wheat Genotype KRL3-4 Provides a New Insight Into Regulatory Mechanisms Associated With Sodicity (High pH) Tolerance

**DOI:** 10.3389/fgene.2021.782366

**Published:** 2022-02-09

**Authors:** Geeta Prasad, Shikha Mittal, Arvind Kumar, Divya Chauhan, Tanmaya Kumar Sahu, Sundeep Kumar, Rakesh Singh, Mahesh C. Yadav, Amit Kumar Singh

**Affiliations:** ^1^ Division of Genomic Resources, ICAR-NBPGR, New Delhi, India; ^2^ ICAR-Central Soil Salinity Research Institute, Karnal, India

**Keywords:** KRL 3-4, transcriptome, sodicity, DEGs, ROS

## Abstract

Globally, sodicity is one of the major abiotic stresses limiting the wheat productivity in arid and semi-arid regions. With due consideration, an investigation of the complex gene network associated with sodicity stress tolerance is required to identify transcriptional changes in plants during abiotic stress conditions. For this purpose, we sequenced the flag leaf transcriptome of a highly tolerant bread wheat germplasm (KRL 3–4) in order to extend our knowledge and better understanding of the molecular basis of sodicity tolerance. A total of 1,980 genes were differentially expressed in the flag leaf due to sodicity stress. Among these genes, 872 DEGs were upregulated and 1,108 were downregulated. Furthermore, annotation of DEGs revealed that a total of 1,384 genes were assigned to 2,267 GO terms corresponding to 502 (biological process), 638 (cellular component), and 1,127 (molecular function). GO annotation also revealed the involvement of genes related to several transcription factors; the important ones are *expansins*, *peroxidase*, *glutathione-S-transferase*, and metal ion transporters in response to sodicity. Additionally, from 127 KEGG pathways, only 40 were confidently enriched at a *p*-value <0.05 covering the five main KEGG categories of metabolism, i.e., environmental information processing, genetic information processing, organismal systems, and cellular processes. Most enriched pathways were prioritized using MapMan software and revealed that lipid metabolism, nutrient uptake, and protein homeostasis were paramount. We have also found 39 SNPs that mapped to the important sodicity stress-responsive genes associated with various pathways such as ROS scavenging, serine/threonine protein kinase, calcium signaling, and metal ion transporters. In a nutshell, only 19 important candidate genes contributing to sodicity tolerance in bread wheat were identified, and these genes might be helpful for better understanding and further improvement of sodicity tolerance in bread wheat.

## Introduction

Achieving global food security in the 21st century seemed arduous due to acute environmental challenges including extreme climatic vulnerability (Fujimori et al., 2019) and persistent land degradation (Subramaniam and Masron, 2021). Salts specifically excess sodium ions affected land degradation distorted nearly 1,128 million ha (Mha) land and causing US$ 27.3 billion economic losses per year (Qadir et al., 2014). Neoteric projections indicated that ∼12 Mha of productive land salinized every year by the dint of natural and anthropogenic processes, and more than half of the total croplands is to be expectedly salinized by 2050 (UNCCD, 2017). Bread wheat (*Triticum aestivum* L.), is the staple food for nearly 2.5 billion of the world population, and constitutes a major share in carbohydrates (55%) and food calorie (20%) consumption in dietary intake (Ramadas et al., 2020). It concedes as a moderately tolerant crop to sodicity (exchangeable sodium percentage, ESP: 35%–50%) stress (Maas, 2019), although with the tolerance, transmogrify with plant species, stress intensity, and developmental stage (Sheoran et al., 2021). Furthermore, an extensive assessment of production loss in wheat cultivated in salt-affected soils revealed 20%–43% yield penalty, only due to adverse soil constraints (Qadir et al., 2014; Chaurasia et al., 2021).

The sodic soil (pH>8.5) is detrimental for mineral elements absorption and impedes with ionic balances in the soils. Globally, more than 30% of the surface land of the earth is covered with high pH soils (Chen and Barak, 1982). Sodic soils having a high level of exchangeable sodium on clay particles tend to develop low porosity with dense structure and high soil strength resulting in restricted root penetration, nutrient uptake, plant water availability, and metabolic activities, thereby adversely affecting crop productivity (Anzooman et al., 2019; Minhas et al., 2019). Previous studies have reported that plants exhibit poor seed germination, seedling survival, stunted shoot and root growth, and decrease in nutrient solubility under sodicity (pH >8.5), consequently, very poor grain yield or fails to reach maturity. The high pH directly affects the mineral element absorption and impedes with ionic balances. Therefore, the elevated soil pH >8.5 is much more detrimental to plants than neutral salts (Yaduvanshi et al., 2012).

Plants have inherent mechanisms to survive under different abiotic and biotic stresses. It is important to unveil the molecular mechanism involved in the regulation of abiotic stress in order to improve the tolerance level of plants. Earlier studies have shown that in adverse conditions, plants cope with the situation through generic stress signal perception and transduction that alters genes responsible for stress and protects plants in the stress condition (Shinozaki and Yamaguchi-Shinozaki, 2007). Past studies revealed that a large number of stress-responsive genes are involved in the stress signaling network. These can be divided into two groups on the basis of their functions and products. The first group include genes involved in protection against cell damage during stress, cell viability such as osmolyte biosynthetic enzymes, antioxidant proteins, and chaperones. In the second group, various transcription factors (TFs) and other parts composed of protein phosphatase and protein kinases are involved (Shinozaki and Yamaguchi-Shinozaki, 2007). Osmotic homeostasis and reactive oxygen species (ROS) scavenging have a critical role in adapting high salt conditions. In addition, iron acquisition, nitrate assimilation, and calcium metabolism could effectively promote a mechanism of tolerance to environment changes in plants (An et al., 2016). Moreover, higher organic acids, such as citric acid accumulation, facilitates tolerance to salinity in soybean (Li et al., 2017), whereas H^+^ secretion in *Arabidopsis* under salt stress might be involved in the regulation of salt tolerance (Shi and Zhu, 2002). Thus, the modulation of ROS scavenging, osmotic homeostasis, and H^+^ secretion jointly helps plants to survive in adverse condition and induce salt tolerance in plants compared with sensitive plants that lack such mechanism of tolerance (Fu et al., 2018).

Until now, research has been primarily focused on saline stress condition, and there are limited studies conducted for understanding of the molecular basis of sodicity stress tolerance (high pH stress) in crop plants (Meng et al., 2017). In our study, a wheat genotype KRL 3–4 that is considered to be one of the most sodicity stress tolerant line was selected to study transcriptional level changes in response to higher pH (Singh et al., 2010). RNA samples collected from wheat flag leaves of plants grown under sodic and normal soils were processed for high-throughput RNA sequencing. GO and KEGG functional enrichment analyses revealed the role of specific DEGs under sodicity stress. Furthermore, a major hub of genes that might have a role in tolerance against sodicity stress were identified. Moreover, bioinformatics analysis unveiled *cis*-acting regions of putative candidate genes that are involved in sodicity stress tolerance.

## Materials and Methods

### Background of Wheat Genotype KRL3-4

The genotype was developed at ICAR-Central Soil Salinity Research Institute, Karnal, India, through conventional breeding method (bulk selection) involving the variety HD 1982 (released in the year 1975 for north eastern plain zone, pedigree: E5557/HD H45) as a female and Kharchia 65 (released in the year 1970 for salt affected lands in all the wheat growing zones, pedigree Kharchia local/EG 953) as a male parent. The selected genotype is tall (140–155 cm) in nature and exceptionally unique in salt tolerance, having pale green foliage and higher early seedling vigor similar to their parent Kharchia 65. It possesses high yielding ability along with higher levels of tolerance to salinity, sodicity, and water-logging compared with their parent Kharchia 65. This conclusion was substantiated by 4 years of observations of the All India Salinity/Alkalinity Tolerance Screening Nursery (SATSN) conducted in different salt-affected agro-ecosystems during 2004–2005 to 2008–2009 (Supplementary Table S1). In the previous literature, Dr. Abdul Mujeeb-Kazi (distinguished scientist and ex program leader of CIMMYT) had already mentioned that Kharchia 65 is the most salt-tolerant bread wheat in the world (Wang et al., 2003).

This germplasm already has been registered (IC408331; INGR09087), in ICAR-National Bureau of Plant Genetic Resources (NBPGR), New Delhi, salt and waterlogging tolerance. The salt tolerance of KRL 3–4 is characterized by better regulation over lower uptake of sodium and highest uptake of magnesium salt-affected soils (Singh et al., 2010). Additionally, it possesses higher tolerance to boron, iron, and aluminum concentrations in the soil solution, and their full potential reflected on highly sodic condition (pH >9.3), where it performs as a halophyte, and it competes with the parent Kharchia 65 in yield as well as in other physiological traits. KRL 3–4 also had the highest grain Ca, Mg, Fe, Zn, and S concentrations compared with most of the Indian cultivars, as it substantiates in an experiment conducted in hostile soil conditions (pH: 4.5–9.5) across the six different agro-ecosystems for 2 years (Khokhar et al., 2018).

### Plant Material and Experimental Design

In our study, a wheat genotype KRL 3–4 was selected to understand the transcriptional changes in response to sodicity stress. The genotype was grown in two concrete lysimeters (dimension: 2 m × 2 m × 1 m) situated at ICAR-Central Soil Salinity Research Institute, experimental farm (29°42′31″N, 76°57′6″E) Karnal, India. The first lysimeter, filled homogeneously with sandy loam soil (Inceptisols, pH: 7.4, ESP:6.3, ECe: 0.9 dS m^−1^, organic carbon: 2.3 g kg^−1^ soil) was considered as control. The other lysimeter was filled homogeneously with incubated synthetic sodic soils formulated by mixing the carbonate and bicarbonate salts in the soils (pH: 9.5, ESP: 74.6, ECe: 1.6 dS m^−1^, organic carbon: 2.1 g kg^−1^ soil). The sowing seeds were surface sterilized in 1% sodium hypochlorite (NaClO^−^) for 10 min and then washed with distilled water several times and planted in the lysimeters. Plants that were grown in normal soil were considered control plants, and other plants grown in the sodic lysimeters (stress intensity: pH-9.5) were considered treated plants. At the anthesis stage, fully matured flag leaf samples were taken from both control and treated plants with two biological replicates. The leaf samples were immediately frozen in liquid nitrogen and stored at −80°C until analysis.

### RNA Extraction and Illumina Deep Sequencing

Total RNA was isolated from the two biological replicates of flag leaves from normal and treated plants (after 12-h exposure to sodicity stress) using RNeasy Plant Mini Kit (Qiagen) according to the instructions of the manufacturer. Equal amounts of total RNA of each two biological replicates were used for the RNA sequencing. Initially, the concentration of RNA was checked by nanodrop and later purity through agarose gel electrophoresis. Furthermore, RNA integrity was estimated by Agilent Bioanalyzer 2100 system (Agilent Technologies Co. Ltd., Beijing, China). Accordingly, the samples with RIN value higher than 9 were used for transcriptome sequencing. After quality control (QC) of the RNA samples, poly (A) enrichment, RNA fragmentation, random hexamer-primed cDNA synthesis, linker ligation, size selection, and PCR amplification reactions were performed to prepare cDNA libraries for each sample. Finally, the qualified libraries fed into HiSeq sequencer according to its effective concentration and expected data volume. Illumina HiSeq 2500 platform was used to sequence libraries, and 150-bp paired end reads were generated.

### Quality Control, Alignment, and Differential Expression Gene Analysis

Raw reads of control and treated samples were evaluated for their quality using FastQC (version 0.11.8) (http://www.bioinformatics.bbsrc.ac.uk/projects/fastqc/). The adaptor sequences and low-quality sequence reads were removed from the data sets using trim galore (version 0.6.2) considering the threshold parameters, including phred score 33, quality score 20, and length 20 (https://github.com/FelixKrueger/TrimGalore/releases). Raw sequences were transformed into clean reads after data processing. The quality of cleaned reads was also checked through base quality score distribution, sequence quality score distribution, average base content per read, and GC distribution in the reads. The obtained high-quality reads were mapped to the reference genome of *Triticum aestivum*, i.e., *IWGSC* v.1.0 (ftp://ftp.ensemblgenomes.org/pub/plants/release-42) using bwa (*version 0.7.5*, http://bio-bwa.sourceforge.net/) with default parameters. These mapped reads were spliced using Cufflinks software based on the reference genome sequence. Quantification of the gene expression levels was estimated as fragments per kilo base of transcript per million fragments mapped (FKPM). Differential expression analysis between control and treated samples was performed using the DESeq R package (1.10.1) (Anders and Huber, 2010). DESeq provides reliability for determining differential expression in digital gene expression data using a model based on the negative binomial distribution. The resulting *p*-values were adjusted using Benjamin and Hochberg’s approach for controlling the false discovery rate. Genes with an adjusted *p*-value <0.05 were found using DESeq and were assigned as differentially expressed. A fold change ≥2 and FDR <0.01 were considered as the thresholds for determining the differential expression of a gene.

### Gene Ontology and Gene Pathway Enrichment Analysis

Gene ontology (GO) enrichment analysis of the DEGs was implemented using topGO R package (https://bioconductor.org/packages/release/bioc/html/topGO.html). The GO terms, i.e., biological process (BP), cellular component (CC), or molecular function (MF) were assigned to the DEGs. Kyoto Encyclopedia of genes and Genomes (KEGG) is a database resource for understanding high-level functions and utilities of the biological systems, such as the cell, organism and ecosystem from molecular-level information, especially large-scale molecular datasets generated by genome sequencing and other high-throughput experimental technologies (http://www.genome.jp/kegg/). KOBAS software (http://kobas.cbi.pku.edu.cn/) was used to analyze the statistical enrichment of differential expression of genes in KEGG pathways. Furthermore, MapMan (version 3.5.1; http://mapman.gabipd.org/web/guest) was used for pathway analysis of DEGs as *p*-value <0.05.

### Alternative Splicing Analysis

Alternative splicing (AS) events for identified DEGs under sodicity stress were determined using an AStalavista web tool (version 3; http://genome.crg.es/astalavista/) with default parameters. The results obtained for all AS events were further analyzed to unravel the molecular mechanisms of AS during exposure of wheat crop to the sodicity stress.

### Identification of Single Nucleotide Polymorphism

The freebayes tool (Garrison and Marth, 2012) and SNP effect predictor (Cingolani et al., 2012) were used to identify and annotate the SNPs, respectively. SNPs and INDEL variants were filtered through bcftools. Based on genomic locations, the variants were annotated as intronic, exonic, 5′UTR, and 3′UTR.

### Candidate Gene Identification

The candidate genes for sodicity stress tolerance were identified on the basis of similarity (blastx with similarity >80% and e-value <0.001) with published or validated genes for their role in sodicity stress tolerance (Singh et al., 2018). The heatmap was made using ggplot2 R package (https://cran.r-project.org/web/packages/ggplot2/index.html). Furthermore, the chromosomal localization of the identified candidate genes was performed using MapChart (Voorrips, 2002), and the 2-kb upstream region was used for the identification of *cis*-acting regulatory elements using PlantCARE database (Rombauts et al., 1999).

## Results

### Sequencing Statistics

In order to understand the mechanism involved at the transcriptional level for sodicity stress tolerance, transcriptome analysis was carried out for control and treated samples of wheat flag leaves. The libraries were sequenced to obtain 150-bp paired-end reads using the Illumina high-throughput sequencing platform. In total, approximately 37.24 Gb of raw data was generated from the two biological replicates of the wheat flag leaf. After trimming, the volume of the total clean reads for all the samples was 36.54 Gb. The clean reads of each sample was more than 8.75 Gb, and the percentage of Q20 bases was ≥97.40%. Sequencing quality distribution was examined over the complete sequence length to detect the sites (base positions) with an unusually low sequencing quality, where incorrect bases may be incorporated at abnormally high levels. Approximately, 97.60%–99.06% of reads are mapped to the wheat reference genome (*IWGSC* RefSeq v1.0, http://www.wheatgenome.org/), whereas GC content varied from 51% to 53% for the control and sodicity stress treated samples (Table 1).

**TABLE 1:** Summary of sequencing and mapping statistics of the bread wheat germplasm (KRL3-4) genotype used in the present study.

	Replicate	Raw data (Gb)	Clean data (Gb)	Mapping %	GC %
Control	Replicate 1	9.28	9.11	98.43	53
	Replicate 2	9.02	8.87	97.61	52
Sodicity stress	Replicate 1	9.98	9.77	99.06	52
	Replicate 2	8.95	8.78	98.99	51

### Identification of Differentially Expressed Genes and Gene Ontology Enrichment

**FIGURE 1:**
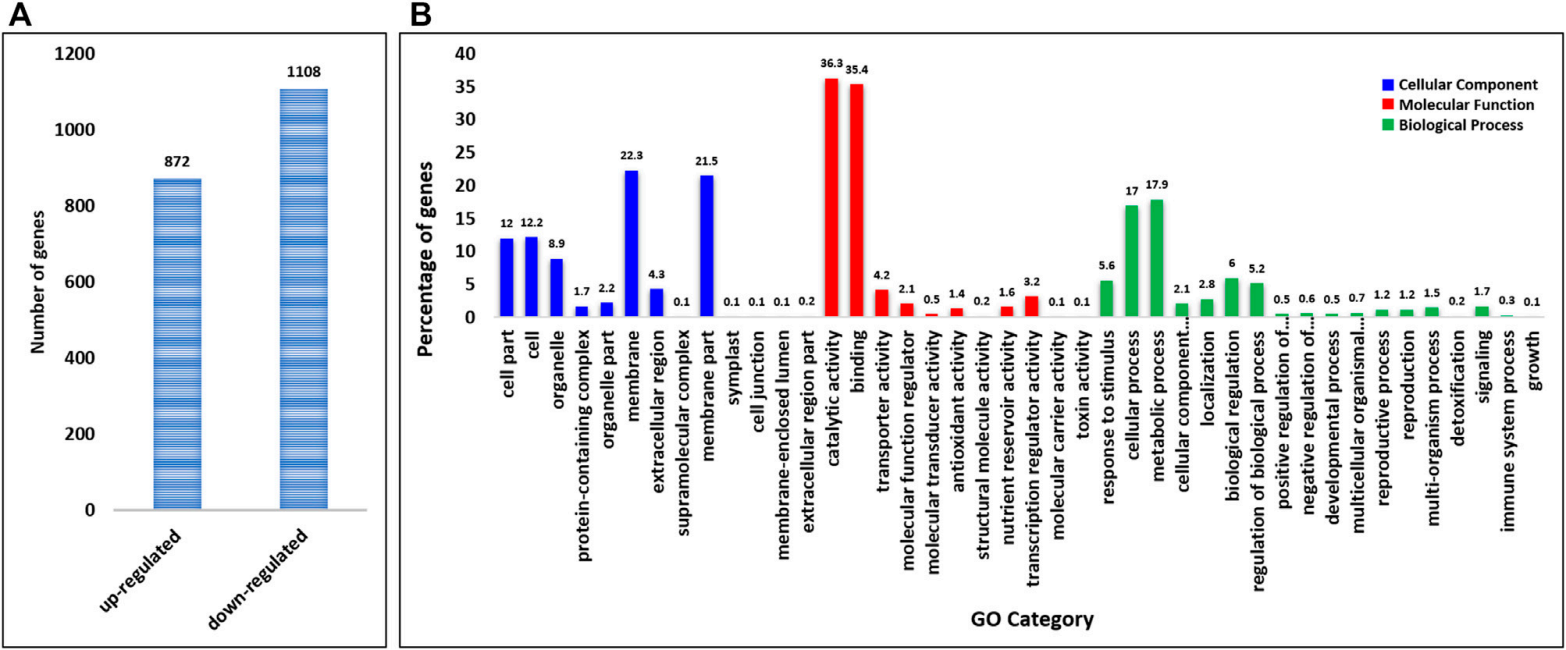
**(A)** Significant up and downregulated differentially expressed genes (DEGs) identified under sodicity stress. **(B)** Gene Ontology (GO) annotation of DEGs in sodicity treated wheat flag leaves summarized in three main categories: biological process (BP), molecular function (MF), and cellular component (CC).

In total, we identified 97,514 differentially expressed genes (DEGs) between control and treated flag leaf wheat samples. The genes with expression level greater than two fold (adjusted *p*-value < 0.05) were considered as significant DEGs under sodicity stress condition with reference to the controlled conditions. At this threshold, there were only 1,980 significant DEGs between control and sodicity stress conditions (Supplementary Table S2). Among these genes, 872 DEGs showed upregulation, and 1,108 showed downregulation under sodicity stress condition (Figure 1A).

Furthermore, GO enrichment analysis was carried out to characterize the main biological functions of DEGs in wheat flag leaves under sodicity stress condition. All the DEGs were annotated into three classes, i.e., BP, MF, and CC. Annotation of DEGs revealed that a total of 1,384 genes were assigned 2,267 GO terms corresponding to 502 BP, 638 CC, and 1,127 MF (Supplementary Table S3). The dominant terms in CC category were “membrane,” “membrane part,” “cell,” “cell part,” and “organelle,” whereas in MF, “catalytic activity,” “binding,” “transporter activity,” “molecular function regulator,” and “molecular transducer activity” were the most dominant GO terms. However, in case of BP, most of the DEGs were classified in “response to stimulus,” “cellular process,” “metabolic process” followed by “biogenesis,” and “localization” (Figure 1B).

As expected, the most enriched BP terms for over-presented DEGs were associated with metal ion transport, response to auxin, water and oxidative stress, transmembrane transport, oligopeptide transport, lipid transport, carbohydrate metabolic process, plant-type cell wall organization, which acted as indicators of significant biological processes underlying the specific sodicity stress responses of plants. The most enriched MF terms for over-presented DEGs were metal ion binding, ATP binding, calcium ion binding, catalytic activity, channel activity, iron and magnesium ion binding, oxidoreductase activity, transferase activity, voltage-gated anion channel activity, etc. Meanwhile, with regard to the over-representation of CC terms among the DEGs, we found extracellular region, membrane, intrinsic to membrane, integral to the membrane, and intrinsic to plasma membrane to be the most enriched.

**FIGURE 2:**
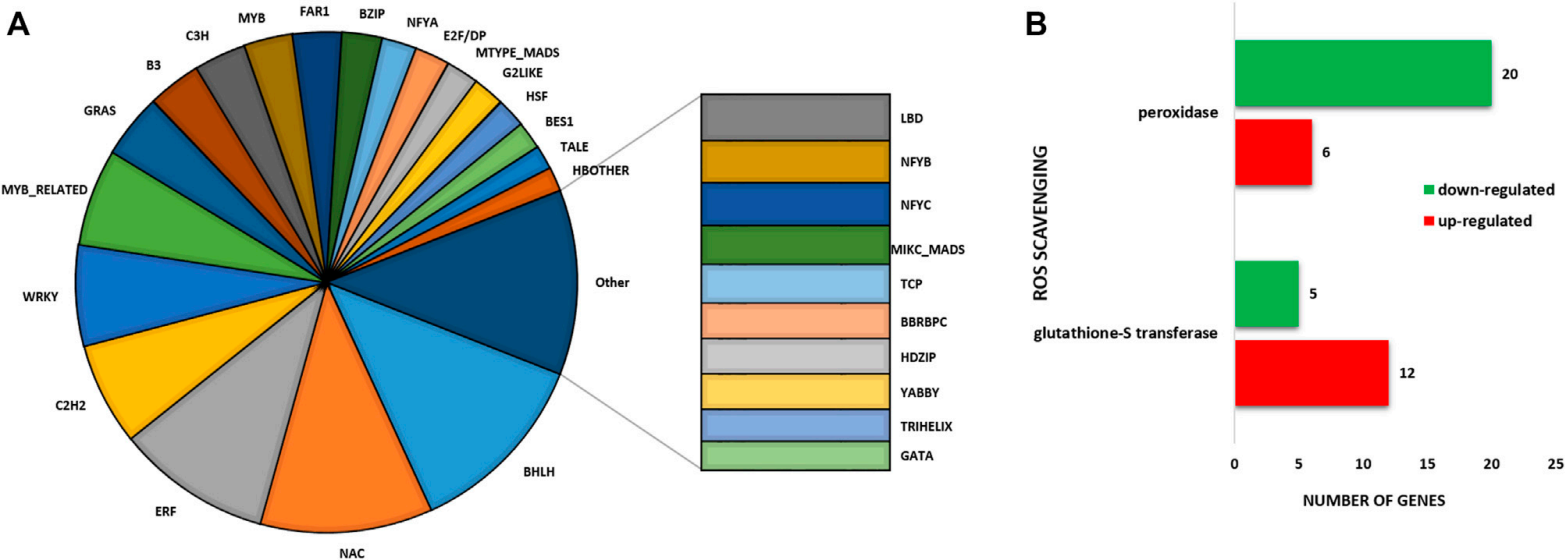
**(A)** Pie chart depicting the involvement of different transcription factors (TFs) in sodicity treated wheat flag leaf samples. **(B)** Bar diagram representing number of the identified reactive oxygen species (ROS) scavenging genes.

Furthermore, a large number of TF genes were found to be differentially expressed under sodicity stress condition. In total, 985 DEGs were encoded for 53 TF families (Figure 2A). The important TF families that were represented by DEGs included *bHLH*, *ERF*, *bZIP*, *NAC*, *MYB-RELATED*, *MYB*, and *WRKY*. Among the TF families, the highest number of genes belonged to the *bHLH* family (109) followed by *NAC* (99), *ERF* (89), *C2H2* (59), *WRKY* (58), *MYB_RELATED* (56), and *GRAS* (37), and so on. We also checked the expression of these TF in control as well as in sodicity treated samples and visualized using heat map. Moreover, we have also identified 37 DEGs encoding for serine/threonine kinase family proteins, out of which 22 showed upregulation, and the remaining 15 showed downregulation under sodicity stress. Similarly, genes encoding for other stress tolerance and signaling-associated proteins were also identified, including 22 genes of calmodulin ion binding and calcium signaling, 11 genes for metal ion transport, 4 genes of ABC transporter, 4 genes of SWEET sugar transporter, and 1 gene encoding potassium transporter. In this study, 43 DEGs appear to protect plant cells from oxidative damage by *ROS* scavenging, such as peroxidase genes (26) and *glutathione S-transferase* genes (17). For peroxidase genes, 6 of the 26 DEGs were upregulated, and the other 20 genes were observed to be downregulated. Besides, the peroxidase gene family, a number of *glutathione S-transferase* genes were found differentially expressed. Most of these genes (12/17) were noticed to be upregulated during sodicity stress (Figure 2B; Supplementary Table S4).

### Kyoto Encyclopedia of Genes and Genomes and MapMan Analysis of Differentially Expressed Genes Under Sodicity Stress

**FIGURE 3:**
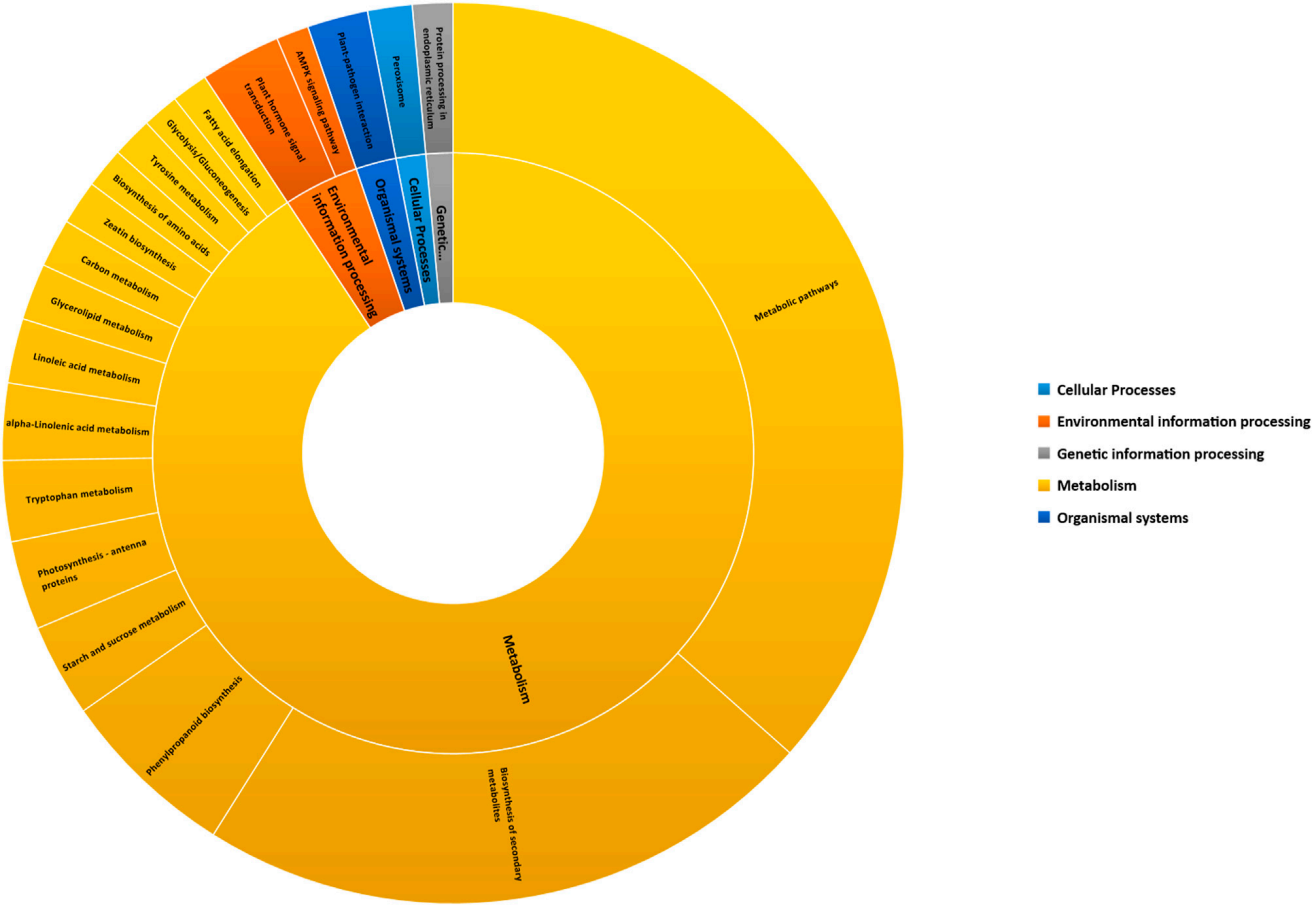
Enriched Kyoto Encyclopedia of Genes and Genomes (KEGG) pathways categorized in top five KEGG pathway categories, i.e., cellular processes, environmental information processing, genetic information processing, metabolism, and organismal systems.

The KEGG pathway analysis was performed to unveil the active biological pathways involved in DEGs under sodicity stress in KRL3-4 flag leaf. KEGG is a database resource for interpretation of large-scale molecular datasets generated by genome sequencing for understanding high-level functions and utilities of the biological system (Kanehisa et al., 2017). The pathway analysis showed that out of 127 KEGG pathways, 40 pathways were observed to be significantly enriched at a *p*-value <0.05 covering the five main KEGG categories of metabolism, environmental information processing, genetic information processing, organismal systems, and cellular processes (Figure 3). The KEGG enrichment pathways indicated that the specific DEGs, found under sodicity stress in flag leaf, were widely enriched in the pathways of metabolic, biosynthesis of secondary metabolites, phenylpropanoid biosynthesis, starch and sucrose metabolism, photosynthesis, and plant hormone signal transduction. According to previous studies, these pathways were found related to abiotic stress tolerance. The list of DEGs involved in 40 KEGG enriched pathway is given in Supplementary Table S5.

**FIGURE 4:**
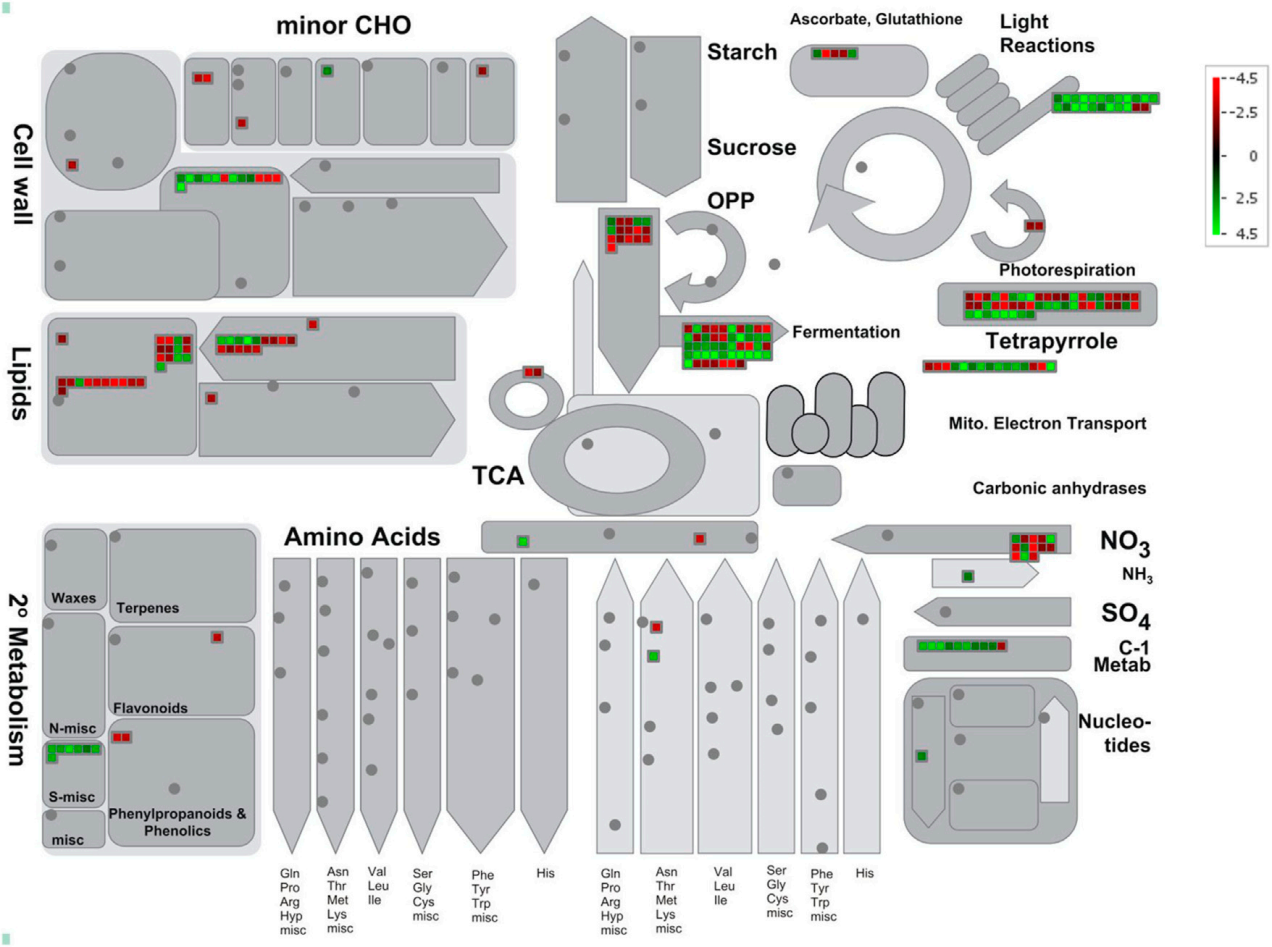
MapMan representing the differential expression of genes involved in metabolism pathway under sodicity stress condition.

The differentially expressed sodicity stress-responsive genes were also visualized using MapMan in order to find out alterations in metabolic pathways during sodicity stress (Supplementary Table S6). The MapMan analysis revealed that lipid metabolism and protein homeostasis, nutrient uptake, etc., were the most enriched pathways under sodicity stress. In lipid metabolism, particularly, the expression of 12 genes involved in lipid degradation, i.e., 9 *phospholipases A1* (*PC-PLA1*) and 3 *phospholipase A2* (*pPLA2-II*) were highly upregulated in response to sodicity stress, whereas the genes related to fatty acid biosynthesis and elongation (11 genes), i.e., *3-ketoacyl-CoA reductase* (*KCR*) and *3-ketoacyl-CoA synthase* (*KCS*) showed mixed expression. *Glycerol-3-phosphate acyltransferase* (*GPAT1-3*) and *acyl-CoA:diacylglycerol acyltransferase* (*DGAT2*), involved in glycerolipid biosynthesis, were found to be up and downregulated, respectively. Similarly, in the case of protein homeostasis, the genes encoding Bowman-–Birk protease inhibitor, pepsin-type protease, amino acid hydrolase was found to be upregulated under sodicity stress. On the other hand, we have also observed the upregulation of *phosphate signaling regulatory protein* (*SPX*), *phosphate transporter 1* (*PHT1*), and *nitrogen limitation adaptation* (*NLA*) genes in nutrient uptake pathway. We identified one gene for *PHT1* (*Traescs2a02g047300*), one for *NLA* (*Traescs5b02g373000*) and seven for *SPX* (*Traescs2a02g169600*, *Traescs2b02g195900*, *Traescs2d02g177100*, *Traescs7a02g376200*, *Traescs7a02g554100*, *Traescs7b02g478000*, and *Traescs7d02g372600*) **(** Figure 4). The cellular overview pathway exposed that the genes encoding for cell wall organization showed mixed expression under sodicity stress. For example, the genes encoding *glutaredoxins* (GRXs) and *arabinogalactan* protein (xylogen) were found highly upregulated while alpha class *expansins* and *glucuronosyltransferase* (GUX) showed downregulation. GRXs are small redox proteins, which use glutathione to catalyze the reduction of disulfide bonds of substrate proteins to maintain cellular redox homeostasis. Furthermore, diverse functions such as transcriptional regulation of defense responses, flower development, oxidative stress response, redox signaling, hormonal regulation, iron homeostasis, and environmental adaptation have been reported for various plant GRXs.

### Alternative Splicing Analysis

**FIGURE 5:**
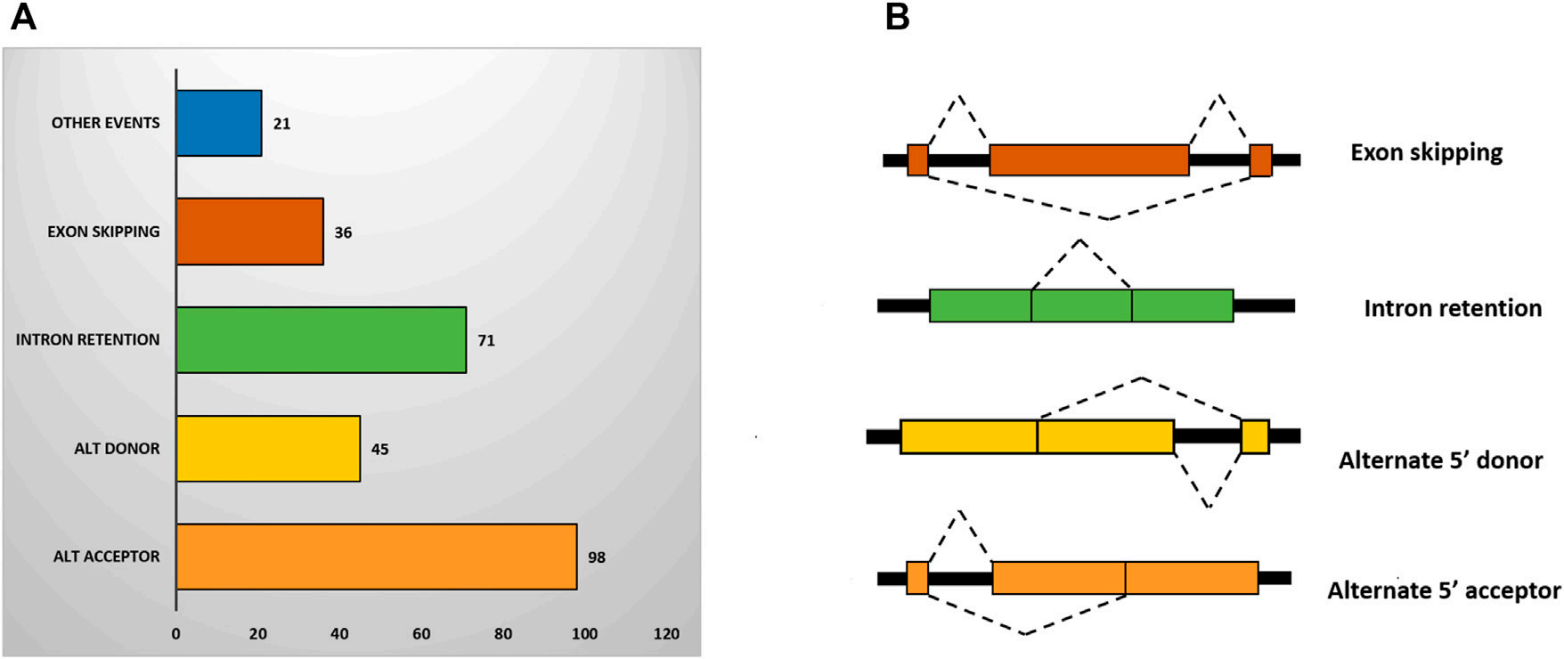
**(A)** Bar chart showing different types of alternative splicing events in DEGs. **(B)** Structure of different types of alternative splicing (AS) events where black box and colored box represent introns and exon, respectively.

Alternative splicing (AS) is an important post-transcriptional mechanism that increases the complexity and diversity of transcriptome and proteome in higher eukaryotes. In plants, AS is found to be involved in a range of functions including plant growth and development, and responses to biotic and abiotic stresses. We have categorized the DEGs into four primary classes of AS events that are retained intron (RI), skipping exon (SE), alternative 5′ splice site (A5SS), and alternative 3′ splice site (A3SS) (Figure 5B). Our study revealed, 271 AS events in the DEGs under sodicity stress. In total, 237 (11.96%) genes out of 1,980 have shown alternative splicing of which the highest number belonged to alternate 3′ acceptor (36.16%) followed by intron retention (26.19%), alternate 5′ donor (16.60%) and exon skipping (13.28%) (Figure 5A). Functional categorization of these alternatively spliced DEGs revealed their involvement in different stress-responsive pathways, including the plant hormone signal transduction, calcium signaling pathway, biosynthesis of secondary metabolites, glutathione metabolism, AMPK signaling pathway, etc. In terms of GO biological processes, these were found to be involved in metal ion transport, response to water, transmembrane transport, lipid and carbohydrate metabolic process, cell wall organization, and abscisic acid-activated signaling pathway, while, the major molecular functions represented by these alternatively spliced DEGs involve abscisic acid binding, metal ion binding, iron ion binding, phospholipase A1 activity, transmembrane transporter activity, etc.

### Polymorphism in the Expressed Sequences

Trait variations are primarily due to sequence level changes in the coding region. The wheat genotype KRL3-4 included in our study represents one of the most sodicity tolerant genotypes of the world. Therefore, one might expect that alignment of the flag leaf transcripts from the control and susceptible conditions against a reference genome of the *cv.* Chinese spring (a relatively susceptible variety) may unravel novel SNPs and INDELs that may have a role in sodicity stress tolerance. A total of 36.64 Gb clean reads was obtained after RNA sequencing of both control and sodicity treated wheat flag leaf samples to discover SNP and INDELs. On an average, one variant was called within every 17 kb in the treating sample and 32 kb in the control sample. In total, 2,35,985 and 1,63,420 polymorphisms (SNPs and INDELs) were identified in the control and treated samples, respectively. In the case of control samples, 39,295,381, and 39,200 SNPs were found as missense, nonsense, and silent mutations, respectively, whereas in sodicity treated wheat flag leaf samples, 26,575, 234, and 26,377 were observed as missense, nonsense, and silent mutations, respectively.

**FIGURE 6:**
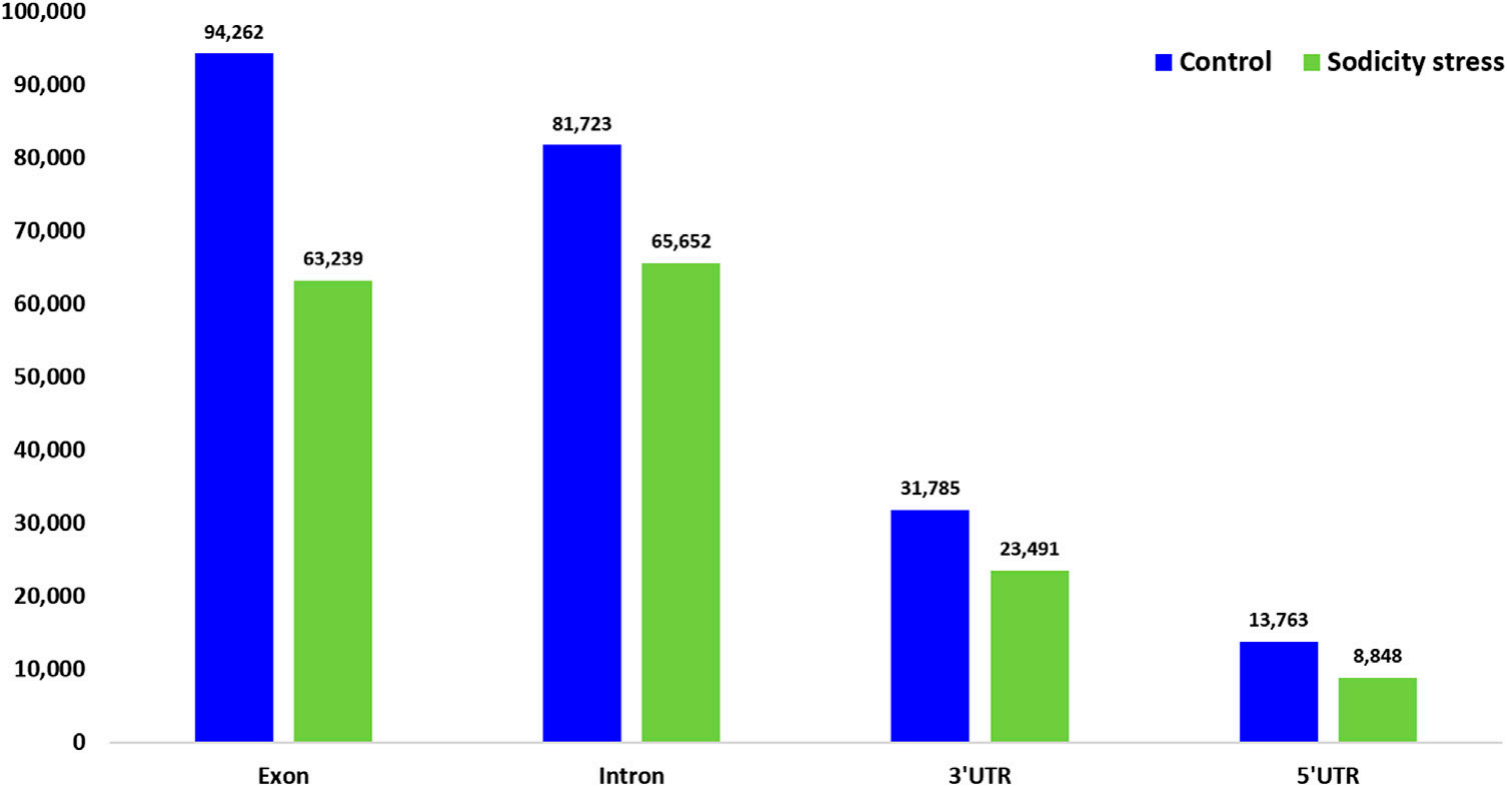
Number of single-nucleotide polymorphism (SNPs) detected in different genomic regions in control and sodicity treated wheat flag leaf using freebayes program.

SNPs and INDELs were mapped to exonic regions that included 8.02% of the total reads (63,239 read counts) in treated and 12.95% (94,262 read counts) in control samples. Moreover, 4.10% of the total reads (32,339) were mapped on 5′UTR and 3′UTR regions in treated samples (Figure 6). In addition, polymorphism due to transitions (Ts) and transversions (Tv) was also determined in each sample. In control, the Ts and Tv were estimated at 7,17,799 and 3,68,641, while in the sodicity treated sample, 4,94,965 and 2,54,817 transition and transversion were detected, respectively. In addition, amino acid substitutions were also identified for SNPs mapped to *IWGSC* wheat reference genome in control and sodicity treated samples. In control sample, the major amino acid substitutions observed were alanine to valine (1,584), alanine to threonine (1,386), and valine to isoleucine (1,004), whereas in treating samples, alanine to valine (1,031), alanine to threonine (902), and valine to isoleucine (709) were noticed to be the major amino acid substitutions.

### Candidate Gene Identification for Sodicity Stress

We have identified 19 sodicity stress responsive genes in our dataset. These candidate genes were identified on the basis of similarity with published validated salt-responsive genes (Table 2). The identified candidate genes encoded for a diverse group of proteins including *LEA*, salt-responsive protein, potassium/sodium cation transporter (HKT), calcium-dependent signaling calcium sensor, *glutathione-S-transferase*, serine/threonine protein kinase, etc. These candidate genes were found to be upregulated under sodicity stress. In terms of GO annotation, these genes were observed to be involved in metal ion binding, hydrolase activity, protein dimerization activity, diacylglycerol O-acyltrasferase activity, DNA binding, polygalacturonase activity, etc. Some candidate genes corresponded to different TF families including *C2H2* (*TraesCS5D02G026300*), *NAC* (*TraesCS2D02G061500*), *WRKY* (*TraesCS5D02G145800*), *AP2/ERF* (*TraesCS5D02G245300*), *ARF* (*TraesCS3D02G276600*), and *bHLH* (*TraesCS5B02G054800*) and showed upregulation in case of sodicity stress compared with control.

**TABLE 2:** List of identified 18 candidate genes for sodicity stress using BLAST search.

Gene ID	Chr	Gene start (bp)	Gene end (bp)	Description	Expression (control)	Expression (sodicity)
TraesCS2D02G061500	2D	25,338,876	25,340,723	NAC domain-containing protein	1.45	14.24
TraesCS2D02G290400	2D	372,695,288	372,696,521	Salt-responsive protein	1.14	12.12
TraesCS3B02G255600	3B	412,595,778	412,597,321	Heat shock factor C1e	1.94	18.68
TraesCS3D02G276600	3D	383,545,476	383,548.591	Auxin-responsive protein	166.37	766.49
TraesCS4D02G102000	4D	80,220,933	80,221,864	HMA domain-containing protein	3.39	38.49
TraesCS4D02G118500	4D	99,161,360	99,162,709	Serine/threonine protein kinase	2.92	69.49
TraesCS5A02G503900	5A	669,584,344	669,585,225	Cold-responsive LEA/RAB-related COR	225.31	990.82
TraesCS5B02G516900	5B	680,671,587	680,672,746	LEA_2 domain-containing protein	4.83	52.68
TraesCS5B02G054800	5B	59,926,012	59,928,225	bHLH domain-containing protein	53.63	228.76
TraesCS5B02G260200	5B	442,821,771	442,822,446	SRC1-clade calcium sensor	1.94	26.13
TraesCS5D02G026300	5D	23,863,473	23,864,186	C2H2-ZF	6.29	246.94
TraesCS5D02G145800	5D	232,453,697	232,455,753	WRKY domain-containing protein	3.87	64.41
TraesCS5D02G245300	5D	353,795,287	353,796,446	AP2/ERF domain-containing protein	1.94	28.48
TraesCS6A02G047600	6A	24,048,539	24,050,177	Peroxidase	1.45	29.12
TraesCS6A02G098500	6A	65,681,325	65,684,847	Chloride channel protein	48.02	308.12
TraesCS6D02G144500	6D	115,043,730	115,046,100	Potassium/sodium cation transporter	40.54	213.00
TraesCS7A02G215100	7A	180,647,622	180,649,835	Fe2OG dioxygenase domain containing	0.49	16.12
TraesCS7A02G516900	7A	701,461,957	701,462,696	Calcium-dependent signaling calcium sensor	0.97	24.82

**FIGURE 7:**
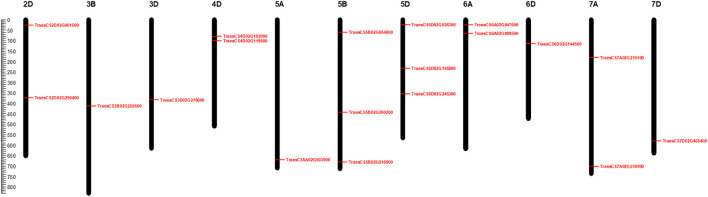
Chromosomal localization of 19 candidate sodicity responsive genes using MapChart.

**FIGURE 8:**
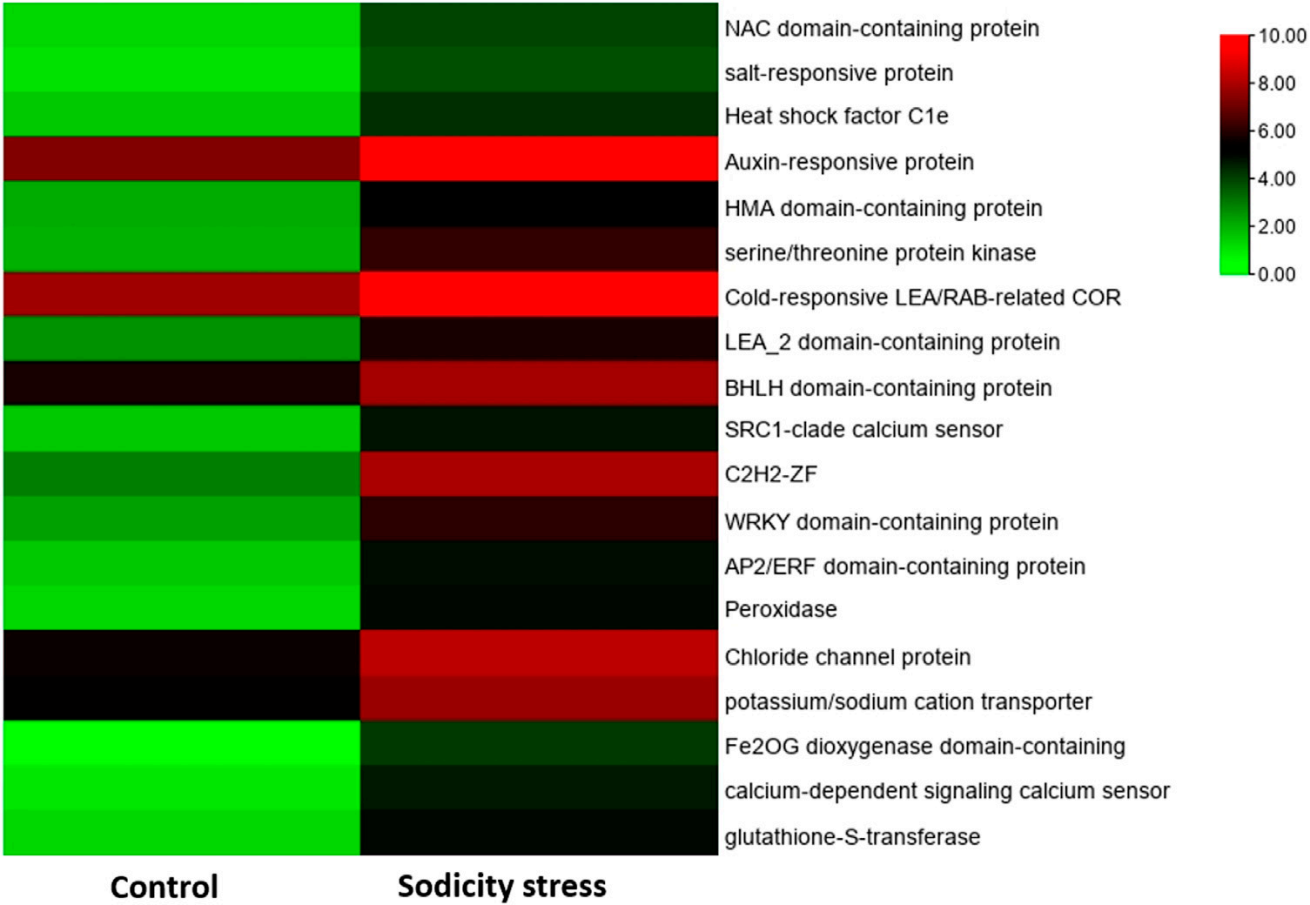
Heat map showing differential expression of candidate sodicity-responsive genes.

Chromosomal distribution analysis showed that 19 identified candidate genes were unevenly mapped in 10 out of 21 wheat chromosomes. On chromosome 5, a total of seven candidate genes were located that represent 36.84% of the entire candidate genes identified. Besides, four genes were located on chromosome 3, whereas on 3B, 3D, and 6D chromosomes, one gene each was located. On the other hand, 2D, 4D, 6A and 7A chromosomes were found to contain two genes each. In contrast, no genes were found in the remaining 11 chromosomes. The sodicity responsive candidate genes were observed to be unevenly distributed among the sub-genomes A, B, and D, with 1, 4, and 10 members representing 5.26%, 21.05%, and 52.63% of the total candidate genes, respectively (Figure 7). The chromosomal distribution of the 19 candidate genes identified in our study is similar to a previous study of bread wheat where the maximum candidate salt-responsive genes were found on D sub-genome than on A and B sub-genomes (Singh et al., 2018) suggesting that sodicity stress controlling genes is preferentially located on the D sub-genome of wheat. The difference in expression patterns of the 19 candidate genes between control and sodicity stress was represented using heatmap (Figure 8). Heat map analysis indicated that nine genes showed contrasting expression between control and sodicity stress, while the rest of the genes were having a mixed expression level. Therefore, this analysis suggests that these genes might have a more certain functional role in sodicity stress tolerance.

### Analysis of *cis*-Regulatory Elements of Identified Candidate Genes Under Sodicity Stress

The *cis*-acting regulatory elements (CARE) have been shown to play a major role in the regulation of gene expression at the transcriptional level in plants. Indeed, these elements are the central component in plant abiotic stress responses since transcription regulation involves association between TFs and, in particular, *cis*-acting regulatory elements (CAREs) of a specific gene (Kaur and Pati, 2016). From the 19 candidate genes identified in this study, the majority belonged to TF categories, which have *cis*-binding motifs that binds to *cis*-elements of the downstream stress-responsive genes and modulate their expression. Identified 19 putative candidate genes were analyzed for their *cis*-regulatory elements using PlantCARE with default parameters (Lescot et al., 2002; Doi et al., 2008). A total of 17 CARE were A-box, ABRE3a, CAAT-box, CCGTCC-motif, CCGTCC-box, CGTCA-motif, DRE-core, G-Box, MTB, STRE, Sp1, TGACG-motif, as-1, AAGAA-motif, MBS, MYC, and TATA-box were observed among identified sodicity responsive candidate genes that have a significant role in stress tolerance. The cis-regulatory elements located in promoter regions of putative 19 sodicity responsive genes with their functional description are given in Table 3.

**TABLE 3:** List of *cis*-acting elements found in candidate sodicity-responsive genes retrieved from PlantCARE with its consensus sequence and transcription factors and their functional description.

S. No	Motif	Consensus/Core-sequence	Transcription factor	Function	References
1	A-Box	CCGTCC	bZIPs	Conserved sequence regulates õ-amylase activity	Sheshadri et al. (2016); Liu et al. (2018); Ijaz et al. (2020)
2	ABRE3a	TACGTG	NAC	*Cis*-acting element involved in abiotic stress and signaling pathway	Zhang et al. (2020)
3	CAAT-box	CCAAT/CAAT	Growth regulating factors (GRFs)	*Cis*-element regulate plant growth and abiotic stress response	Huang et al. (2021)
4	CCGTCC- motif	CCGTCC	ERFs	Involve in ABA response and has a role in meristem specific regulation	Li et al. (2020)
5	CCGTCC-box	CCGTCC	Iron-responsive element (IRE)	Is related to meristem specific activation	Wang et al. (2016)
6	CGTCA-motif	CGTCA	MeJA	*Cis*-regulatory element involved in the MeJa response	Xing et al. (2020)
7	DRE-Core	GCCGAC	DREB/ERFs	It mediates tolerance against biotic and abiotic stress and has a role in signaling	Zhou et al. (2010)
8	G-Box	CACGTG	bZIPs, bHLH and NAC	Ubiquitous *cis*-acting element responses to various stress, i.e., drought, high salt, cold/freezing	Qian et al. (2021); Shah et al. (2021)
9	MYB	CAACAG	MYB-TF family	Plays a role in developmental processes and stress response	Chen et al. (2018); Hao et al. (2021)
10	STRE	AGGGG	ZnF	Regulates pathways initiated by abiotic and biotic stresses	Estruch (2000)
11	Sp1	GGGCGG	HD-ZIP	Putative role in drought and salinity stress	Pandey et al. (2016)
12	TGACG- motif	TGACG	MeJA	JA-responsive element	Liao et al. (2017)
13	as-1	TGACG	bZIPs, MeJA	Involvement in stress and salicylic acid-responsive element	Banerjee et al. (2015)

## Discussion

Sodicity stress is one of the serious abiotic stresses that causes severe threat to global food security, leading to desertification of land affecting crop yield and quality (Singh et al., 2015; Li et al., 2018). Most plants, including wheat and other cereal species can barely persist in sodicity soils due to high-pH toxicity, low osmotic potential of soil, and imbalanced organic composition which lead to inhibition of plant growth resulting into reduced crop productivity (Msimbira and Smith, 2020). Therefore, it is important to understand detailed molecular mechanisms underlying sodicity stress tolerance in wheat. The comprehensive understanding of cellular pathways and genes involved in salinity stress response can contribute to the development of high yielding sodicity stress tolerant wheat cultivars.

**FIGURE 9:**
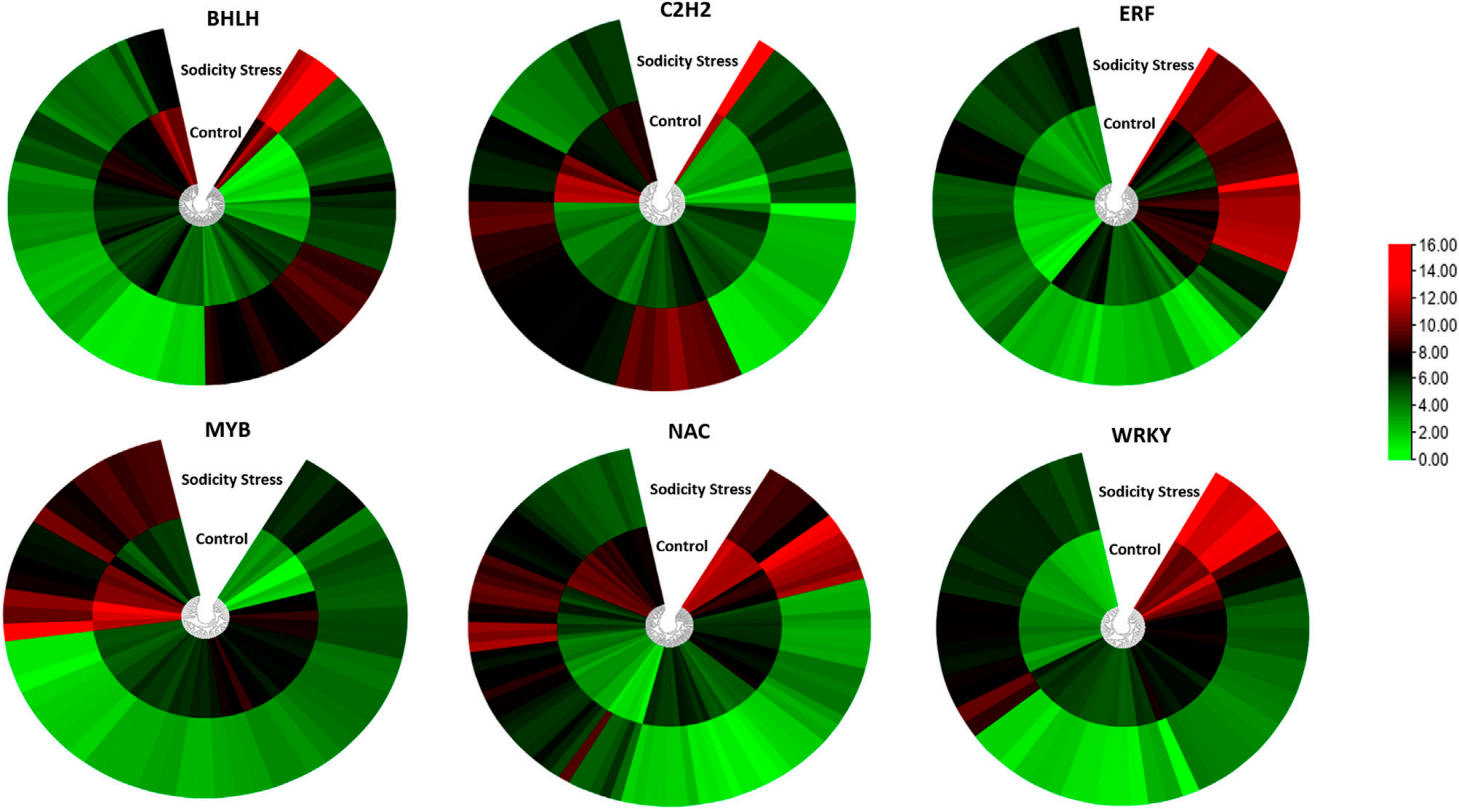
Heatmap representing differential expression of sodicity-responsive TF families in wheat leaf samples.

Transcriptomic approach has emerged as a very effective tool to unravel key pathways/genes responsible for abiotic stress tolerance in plants (Elamin et al., 2019). So far, there is only one report of transcriptomic analysis of wheat in response to sodicity stress. Hence, we conducted a high-throughput RNA sequencing of wheat flag leaves RNA samples under sodicity stress conditions. The statistical analysis revealed 1,891 DEGs in response to sodicity stress. The GO analysis revealed that the DEGs were related mainly to the TFs, serine-threonine kinase protein, metal ion transporters and ROS scavenging under sodicity stress. Similarly, a previous study of wheat revealed higher ROS scavenging ability as a common feature in defending salt-alkali stress (Meng et al., 2017). In our study, many TF families were differentially expressed under sodicity stress, for instance, *bHLH*, *NAC*, *ERF*, *WRKY*, *C2H2*, *MYB*, etc., were highly enriched in the sodicity stress treatments (Figure 9). Past studies have reported involvement of these TFs in response to environmental stresses, such as cold, salinity, drought, iron deficiency, and low nitrogen (Wang et al., 2017; Mittal et al., 2018; Guo et al., 2021b; Wang et al., 2017; Guo et al., 2021a). The TF families such as *bHLH*, *NAC*, *WRKY*, *ERF*, and *MYB* were highly enriched in *Tamarix hispida*, *Ziziphus acidojujuba*, and *Glycine soja* roots under sodicity stress condition (Ge et al., 2011; Wang et al., 2014; Guo et al., 2017). Conversely, studies on different plants such as, *Arabidopsis*, rice, chickpea, and tomato demonstrated that high expression of *NAC* transcription factors support in attaining sodicity stress tolerance by regulating stress-responsive genes and enhanced physiological activities (Huang and Dai, 2015). Additionally, a zinc finger protein, is related to the osmotic stress tolerance in *Arabidopsis* (Muthamilarasan et al., 2014; Ma et al., 2016). Similarly, the overexpression of *OsWRKY8* increased tolerance to osmotic stress in *Arabidopsis* by modulating ABA-independent responsive gene (Yu et al., 2010). The high enrichment of these TF families in sodicity environment suggests their involvement in the regulation of sodicity tolerance.

In addition, we also identified 37 DEGs encoding *serine–threonine protein kinases* (*STPK*). Phosphorylation by *STPK* is one of the primary signaling events that occur in response to environmental stresses (Ichimura et al., 2000). Various studies have depicted the role of *STPK* in abiotic stresses (salinity, drought, and cold), in many crops including *Arachis hypogea* (Rudrabhatla and Rajasekharan, 2002), alfalfa (Chinchilla et al., 2003), *Arabidopsis* (Umezawa et al., 2004), *Oryza sativa* (Diédhiou et al., 2008), and *Triticum aestivum* (Mao et al., 2010). For example, in *Arabidopsis*, upregulation of *AtSnRK2.8* enhanced drought tolerance (Umezawa et al., 2004) and high expression of *OsSAPK4* significantly increased the salinity tolerance of transgenic plants (Diédhiou et al., 2008). In wheat, ectopic expression of *TaSnRK2.4* leads to extended primary roots, late seeding development, and improved tolerance to abiotic stresses in *Arabidopsis* (Mao et al., 2010). The fact that a large number of *STPK* genes were highly induced under sodicity condition suggested their involvement in signal transduction under salinity stress.

In plants, metal ion transporters are important proteins involved in ion homeostasis under sodicity stress conditions. Our study revealed differential expression of transporter encoding genes such as potassium transporter, *ABC* transporter, *SWEET* sugar transporter, and so on, in sodicity treated wheat flag leaf. The sugar metabolism, synthesis of amino acids, and enhanced activity of transmembrane kinase activity are important players in conferring enhanced level of salt stress tolerance in plants and sugar accumulation that provides energy for *ABC* transport regulation where amino acids are the key precursors for the synthesis of some important secondary metabolites (Jia et al., 2020). Moreover, our study also revealed that transmembrane transport protein was enriched under sodicity stress, such as *ABC* transporters, phosphate transporters, transmembrane amino acid transporters, and transmembrane anion transporters. It is evident that the tolerance of wheat to sodicity stress was achieved by increasing the activities of transmembrane proteins and ion channels to exchange ions and small molecules.

KEGG pathway analysis was performed to identify related pathways for 1,891 DEGs that were enriched in this study. Our study revealed the involvement of phenylpropanoid biosynthesis, biosynthesis of secondary metabolites, and plant hormone signal transduction pathways in sodicity condition. In plants, multiple studies have reported that these pathways are significant in abiotic and biotic stress response (Goyal et al., 2016). Phenylpropanoid pathway with the third highest number of genes is a metabolic pathway accountable for the production of different plant secondary metabolites having roles in development and stress-related processes (Baxter et al., 2014).

### MapMan Pathways Expressed in Wheat Flag Leaf Under Sodicity Stress

In our study, 604 DEGs were mapped using the MapMan tool to analyze the impact of sodicity stress on wheat flag leaves. Our results suggested that in response to sodicity stress, a large number of genes were found to be associated with lipid metabolism, protein homeostasis, cell wall synthesis, and modification. In addition, transcriptional regulation of defense responses, flower development, oxidative stress response, redox signaling, hormonal regulation, iron homeostasis, and environmental adaptation were also reported for various plant *GRXs*.

Lipids are structural, storage, and signaling molecule components in plant cells. They are the principal constituents of plant membranes and the cuticle (Harwood, 1980). In this study, Mapman analysis showed upregulation of various lipid metabolism genes including 12 genes encoding for *phospholipase*, 9 genes encoding for *phosphocholine phosphatase*, and 1 gene encoding for *glycerol-3-phosphate acyltransferase* under sodicity stress. All three together are involved in the synthesis of *triacylglycerol* (*TAG*) that is an important component of lipids in cell membranes. Prior studies have shown that different stress conditions induce TAG production in vegetative tissues leading to upregulation of several key genes involved during TAG biosynthesis. Abscisic acid (ABA), a plant hormone, accumulates under stress and functions as a signaling molecule to regulate plant development and metabolic pathways. A transcription factor, ABSCISIC ACID INSENSITIVE 4 (ABI4), that is a key component in the ABA signaling pathway was upregulated under stress conditions and was observed to bind the DGAT1 promoter, which induces its expression in *Arabidopsis* (Lu et al., 2020). Therefore, this important observation supports the fact that salt stress generally disturbs the integrity of the plasma membrane and the high level of TAG might be contributing to salt stress tolerance in KRL3-4 by maintaining integrity of plant membranes. Another component involved in lipid metabolism is 3-ketoacyl-CoA synthase (11), which was found to be downregulated under sodicity condition. Earlier research showed that cuticular wax was found to be associated with plant resistance in response to abiotic stress. *3-Ketoacyl-CoA synthase* (*KCS*) catalyzes the biosynthesis of very-long-chain fatty acid (VLCFA) wax precursors. *3-Ketoacyl-CoA* (*KCS*) synthase encoded by three salt-responsive genes (*LOC_Os02g11070*, *LOC_Os05g49900*, and *LOC_Os02g56860*) have a role in wax biosynthesis (Yuenyong et al., 2018). Furthermore, a recent study in navel orange showed that a novel KCS family gene named *CsKCS6* regulate the production of fatty acid precursors involved in wax synthesis and delivers tolerance of transgenic *Arabidopsis* plants in abiotic stress. Their experiment demonstrates that the expression of *CsKCS6* significantly increased the amount of VLCFAs in the cuticular wax in transgenic *Arabidopsis* plants. The mechanism of *KCS6*-like genes to influence cuticle permeability in transgenic plants involves ectopic expression of *CsKCS6*, which decreased the amount of primary alcohols and aldehydes suggesting that wax biosynthetic genes might be suppressed by ectopic expression of *CsKCS6* in transgenic plants. Hence, a significant decrease in leaf chlorophyll permeability, water loss rate, ion leakage, increased root length, and survival rate were observed in the transgenic plants under drought and high salt stress indicating that ectopic expression of *CsKCS6* can reduce the cuticle permeability and enhance plant tolerance to drought and high salt (Guo et al., 2020). Furthermore, two *peroxygenases*/*caleosin* encoded by *CLO/PXG*-like genes were also found, showing downregulation during sodicity-induced condition in our analysis. *Caleosins* are a calcium sensor carrying one EF-hand calcium-binding motif. A transcriptomic study on fungus has shown that the *CLO/PXG*-like genes have complex patterns of developmental and tissue-specific expression and exhibited response toward biotic and abiotic stresses and play key role in lipid metabolism, signaling, reproduction, and pathogenesis (Rahman et al., 2018). In another study on rice, *caleosin family protein 5 (OsClo5)* or *peroxygenase (PXG)* modulates salt stress tolerance *via* calcium-dependent membrane associated hemoprotein that catalyzes the *PXG* reaction (Blée et al., 2012) indicating its potential roles in enzymatic activities and Ca^2+^ signaling in plants. *OsClo5* interacts with *OsDi19-5* and inhibits transcription of *OsUSP* and *OsMST* and that activity leads to the enhanced sensitivity of rice seedlings in response to salt stress compared with the wild-type, where *OsClo5* with T-DNA knockdown mutation distorted heterodimer formation with *OsDi19-5* and promotes the weak transcriptional repression effect on the two target genes, i.e., *OsUSP* and *OsMST*. This confers the reduced sensitivity to salinity or the enhanced tolerance to salt stress in rice seedlings in comparison with wild-type. Thus, *OsClo5* negatively affected salt stress tolerance in rice in cooperation with the transaction repression control of *OsDi19-5* to two target genes *OsUSP* and *OsMST* in rice (Jing et al., 2021).

In addition to lipid metabolism, many DEGs encoding the Bowman–Birk protease inhibitor (BBI), such as *TraesCS3D02G034200*, *TraesCS3A02G046000*, *TraesCS3A02G046100*, *TraesCS1D02G022000*, *TraesCS5A02G484800*, *TraesCS5B02G498100*, and *TraesCS5D02G498300*, showed upregulation. The Bowman–Birk inhibitor (BBI) is one of the subfamilies of serine protease inhibitors. It has been reported to function in controlling abiotic stresses, such as salinity and drought stresses (Prakash et al., 1996). In a previous study on wheat, a salt-responsive gene *WRSI5* that contains a Bowman–Birk domain was found responsible for tolerance to salt stress. It was characterized from the salt-tolerant cultivar Shangrong No.3 (SR3) and salt-susceptible cultivar JN177. The *WRIS5* gene functions as trypsin inhibitor as it exhibits a high level of trypsin-inhibiting activity, which signifies that BBI suppressed the trypsin activity, thus, promoting elevated *WRSI5* expression level in SR3 roots in salt, drought, or oxidative stress. Furthermore, the SR3 allowed limited transport of K^+^ than Na^+^ from root to shoot leading to low Na^+^ concentration in SR3 leaves resulting in increased leaf growth rate. In addition, overexpression of *WRSI5* in *Arabidopsis* improves seedling growth at 150 mM NaCl condition, suggesting that *WRSI5* controls plant growth rate or long-distance Na^+^ transport in SR3 plants under salt stress (Shan et al., 2008). Similarly, BBI family genes expressed in our study might function in sodicity tolerance.

Furthermore, our study showed upregulation of 13 *glutaredoxin* (*GRX*) genes in response to sodicity stress. The *GRX* family proteins are involved in various cellular functions, such as redox regulation and protection under abiotic and biotic stresses (Lillig et al., 2008). A recent report showed overexpression of *glutaredoxin* (*OsGrx_C7*) ubiquitously expressed in rice including root and shoot, thus indicating positive response in salt induced stress. However, silencing of this gene exhibited increased sensitivity of rice plants to salt stress. Furthermore, *OsGrx_C7* was suggested to be a positive regulator of salt tolerance by reinforcing the expression of transporters employed in Na^+^ homeostasis in overexpressing rice plants (Verma et al., 2021). Therefore, *GRX* genes found in our study can be considered to play a role in sodicity tolerance through Na^+^ homeostasis. Additionally, eight downregulated DEGs encoding for α-*expansins* were also identified in our study. *Expansins* are key regulators of cell wall formation and required for cell enlargement under various environmental stresses (Wu and Cosgrove, 2000). Several studies have shown that the expression of *expansin*-encoding genes are induced under high salt and tends to promote salt tolerance in plants (Qiu et al., 2014). *Expansin* is a superfamily of plant proteins and comprised of four families, such as α-*expansin*, β-*expansin*, *expansin*-like A, and *expansin*-like B. The α and β-*expansin* proteins entail cell wall loosening activity and are involved in cell expansion and plant developmental processes. Recently, transgenic tobacco plants, including α*-expansin 4* (*EXPA4*) overexpression and RNA interference (RNAi) mutants were assayed for their tolerance to abiotic stress. The study revealed that transgenic plants with altered *EXPA4* demonstrated that RNAi mutants have increased hypersensitivity to salt and drought stress and vice-versa in overexpressed tobacco lines, consequently, leading to lower cell damage, higher fresh weight, soluble sugar, proline accumulation, and induces several stress responsive gene expression. Therefore, *EXPA4* indicated its involvement in salt and drought stress response *via* changes in the expression levels of some stress-responsive genes (Chen et al., 2018). Moreover, downregulation of arabinogalactan proteins (*AGPs*) was also seen in our study. *AGPs* were seen involved in abiotic stress response such as salinity regulating cell wall expansion. Several studies have reported downregulation of *AGPs* in response to salt treatments. For example, in tomato, different *AGPs* were strongly repressed in salt-treated tomato roots (Ouyang et al., 2007). Similarly, two rice *OsFLA* (*OsFLA10/18*) and two wheat *TaFLA3/4* genes were significantly downregulated under salinity (Faik et al., 2006; Ma and Zhao, 2010). *AGPs* also act as a sodium carrier through the mechanism of vesicle trafficking (Garcia de la Garma et al., 2015). Moreover, the tobacco (*Nicotiana tabacum* L.) cells cultured in saline medium, which were deficient in *AGPs* on the plasma membrane, demonstrates a reduced rate of cell enlargement and a decreased cell wall extensibility. However, non-saline tobacco cells showed higher upregulation of *AGPs* in salt-stress indicating an important increase rate in *AGP* diffusion through a much more highly porous pectic network leading to improved cell wall formation (Zhu et al., 1993; Lamport et al., 2006). This mechanism may contribute to sodium homeostasis during salt adaptation to high saline concentrations (Olmos et al., 2017). Overall, our study revealed diverse classes of genes as key mediator of salinity stress response and could be further validated using functional genomics tools to uncover their exact role in salt tolerance.

### Nutrient Uptake Regulation in Bread Wheat Germplasm Under Sodicity Stress

Sodic soil is known to decrease the availability of nutrients such as phosphorus required for plant growth and development (Dotaniya and Meena, 2015). In plants, the primary source of phosphorus is inorganic orthophosphate (Pi), but in sodic soil, it forms an insoluble compound with calcium ions and becomes inaccessible (Péret et al., 2011). As a result, a deficiency in Pi stimulates a set of plant adaptive responses with the purpose of decreasing Pi usage and increasing Pi uptake and recycling *via* upregulation of regulators and transporters involved in P-homeostasis (Ticconi and Abel, 2004; Desnos, 2008; Lin et al., 2009; Rouached et al., 2010). Various genes related to low phosphorus stress have been identified in different plant species (Rausch and Bucher, 2002; Jain et al., 2007; Wang et al., 2011). For example, *phosphate transporter 1* (*PHT1*) gene family participates in the uptake of Pi from the soil (Muchhal et al., 1996; Mudge et al., 2002; Shin et al., 2004; Jia et al., 2011). In our study, we have observed one *PHT1* (*Traescs2a02g047300*) gene, which showed upregulation under sodicity stress. *PHT1* transporters are found to be involved in the uptake of phosphate from the soil and transport it to the shoot and mycorrhizal symbiotic interface. In *Arabidopsis*, rice, and maize, *PHT1* expression is strongly upregulated during phosphorus starvation (Smith and Read, 2008; Huang et al., 2011). Similarly, in wheat also, two *PHT1* members, i.e., *TAPHT1.1* and *TaPHT2.1*, overexpressed in Pi-deficient condition (Shukla et al., 2016). Our study also revealed the upregulation of seven *phosphate signaling regulatory protein* (*SPX*), mapped to the 2A, 2B, 2D, 7A, 7B, and 7D chromosomes (*Traescs2a02g169600*, *Traescs2b02g195900*, *Traescs2d02g177100*, *Traescs7a02g376200*, *Traescs7a02g554100*, *Traescs7b02g478000*, *Traescs7d02g372600*). These genes perform multiple functions in plant tolerance to phosphorus starvation and plays a vital role in sensing the concentration of phosphorus in cytosol (Duan et al., 2008; Wild et al., 2016). During Pi starvation, *SPX1* dissociates with the *phosphate response 1* (*PHR1*) TF. *PHR1* controls many Pi related including miRNA399, which targets *phosphate 2* (*PHO2*). Furthermore, *PHO2* reduction leads to *PHT1* and *PHO2* accumulation and, last, an increase in the plant capacity to uptake Pi and translocate it to shoots. Various genes participate in the proper functioning of this signaling pathway, such as *SUMO E3 ligase SIZ1*, *phosphate transporter traffic facilitator* (*PHF1*) and *nitrogen limitation adaptation* (*NLA*) (Lin et al., 2013). According to our MapMan results, we have also found one gene encoding *NLA* (*Traescs5b02g373000*), highly upregulated under sodicity stress. *NLA* functions of the plasma membrane to direct the degradation of PHT1s is a process necessary for Pi uptake ability of plants (Lin et al., 2013). Hence, we can conclude that although we have observed the expression of many transporter proteins and their respective genes under sodicity stress, the physiological role of Pi transporters in response to sodicity stress is still limited. Understanding the molecular mechanism involved in the regulation of phosphate transporters is very important, due to the constant lowering availability of phosphorus reserves in nature and will facilitate the development of more efficient Pi-utilizing plants.

### *Cis*-Regulatory Elements Identified in Candidate Sodicity Stress-Responsive Genes

Plants undergo adaptation during stress conditions by triggering a network of signaling cascade that leads to activation of a series of target gene expression. Studies have shown that several TFs, when binding to specific *cis*-elements, act as molecular regulators of gene expression during stress responses (Gujjar et al., 2014). PlantCARE analysis revealed recognition of many *cis*-elements in genes expressed in response to sodicity stress (Table 3). For instance, A-box, CAAT-box, CGTCA-motif, G-Box, STRE, TGACG-motif, as-1, TATA-box were present in all 19 genes followed by MYB and MYC observed in 18 genes, whereas CCGTCC motif, CCGTCC-box, DRE core, and Sp1 were seen in 17 genes, and ABRE3a, AAGAA-motif, MBS were located in 13 genes. Previous studies have reported that these *cis*-elements in many genes are expressed in response to abiotic stresses. For example, “CGTCA-motif” was reported in drought stress-responsive genes of trifoliate orange and suggested to play crucial role in drought response *via* jasmonic acid-mediated signaling (Xiong et al., 2020). Similarly, “G-box” *cis*-acting DNA regulatory element are recognized by the *bZIP* and *bHLHs* family TFs to mediate responses toward abiotic and biotic stresses (Qian et al., 2021). Prior studies have shown that *bZIP* and *bHLHs* family TFs bind to “G-box” in the promoter of cold, drought, and salinity-responsive genes in *Arabidopsis* and other plant species (Liu et al., 2008; Shah et al., 2021). In wheat, a *TabHLH13* gene that responds *via* “G-box” elements were upregulated under salt stress (Fu et al., 2014). Another important *cis*-element, i.e., “STRE” is recognized by *zinc-finger DNA binding* TF (Estruch, 2000). “STRE” is functional in both orientations (“CCCCT” or “AGGGG”). A study conducted on yeast showed that the “STRE” *cis*-element located in the promoter of *CTT1* and *DDR2*, a DNA damage-responsive gene mediates transcriptional induction in response to different stress conditions (Kobayashi and McEntee, 1993). In contrast, “TGACG-motif,” with consensus sequence “TGACG” is targeted by MeJA family TFs as it acts as JA-responsive element (Liao et al., 2017). In rice, two “TGACG-motif” (JA-responsive element) *cis*-elements were found in the promoter region of *OsTMP14* that is related to stress tolerance. Subsequently, activation sequence-1 (“as-1”) with consensus sequence “TGACG” situated in all identified genes showed the role in abiotic stress and salicylic acid responsiveness (Banerjee et al., 2015). In the current study, two *cis*-acting elements (“*MYB*” and “*MYC*”) were located in 18 candidate genes that might be involved in sodicity stress tolerance. The *MYB* cis-element with consensus sequence “CAACAG” mostly interacts with MYB TFs. In past reports, overexpression of the wheat MYB TFs, such as *TaMYB30-B*, *TaMYB33*, *TaMYB56-B*, and *TaSIM*, import tolerance to salt and drought stresses in transgenic *Arabidopsis* (Yu et al., 2017), whereas *MYC cis*-element with consensus sequence “CATGTG” functions in salt and drought stress response through interaction with *bHLH* and *NAC* family TFs. The role of “*MYC*” has also been seen in drought tolerance through the dehydration-inducible expression of *EARLY RESPONSIVE TO DEHYDRATION STRESS 1 (ERD1)* gene in *Arabidopsis*. Since *ERD1* promoter contains the “CATGTG” motif that interacts with NAC, which specifically binds to the MYC-like sequence “CATGTG,” the expression of NAC TFs genes, such as *ANAC019*, *ANAC055*, and *ANAC072*, was induced by drought, high salinity, and abscisic acid (Tran et al., 2004).

*Dehydration-responsive element* (*DRE*) core having “GCCGAC” consensus sequence is found in promoters of various environmental stress-responsive genes. DREB TFs binds at “DRE-core” (Liu et al., 1998). In *Arabidopsis*, overexpression of *DREB1A* promotes enhanced expression of downstream drought stress-responsive genes (Liu et al., 1998). In addition, DREB proteins also have a role in phytohormone signaling pathway such as abscisic acid, salicylic acid, jasmonate acid, ethylene, and gibberellic acid (Choi et al., 2002). “Sp1” is another *cis*-element present in 17 identified candidate genes. *Homeodomain leucine zipper* (*HD-ZIP*) TFs targets the consensus sequence “GGGCGG” of “Sp1” for abiotic stress response. The presence of “Sp1” in the promoter region of banana *HD-ZIP* genes have shown its participation in various functions and were found to be upregulated by drought and salinity stress (Pandey et al., 2016).

### Importance of Polymorphism in the Expressed Sequences

SNP identification may have a critical role in conferring sodicity stress tolerance in wheat flag leaf. We noticed 39 SNPs mapped to the important sodicity stress-responsive genes associated with various pathways, such as ROS scavenging, serine/threonine protein kinase, calcium signaling, and metal ion transporters) that are known to have a role in abiotic stress responses. From these 39, a total of 29 SNPs were mapped to ROS-scavenging genes that were differentially expressed during sodicity stress condition, thus, stimulating stress tolerance through glutathione metabolism and the biosynthesis of secondary metabolite pathways in wheat. In addition, SNPs were also mapped to highly upregulated metal ion transport, AMPK signaling, and ABC transport activity (Table 4). These results suggest that besides variation in expression pattern, sequence level polymorphism in key abiotic stress genes may also have a role in conferring enhanced level of sodicity stress tolerance in KRL 3–4.

**TABLE 4:** Polymorphisms identified in the DEGs by comparing with IWGSC Chinese spring (RefSeq v1.0) reference genome.

Annotation	Gene ID	Chr	Position	Reference genome	KRL3-4
ROS scavenging	TraesCS1A02G186600	1A	337,677,633	C	T
			337,677,955	A	C
	TraesCS1B02G113700	1B	133,076,406	G	–
	TraesCS2B02G126200	2B	94,223,974	T	C
			94,224,203	G	A
	TraesCS3A02G297100	3A	531,701,836	GTCGGAAGTGAGT	A
				CCCTT	
			531,701,361	C	GAAA
			531,701,367	AAAG	C
			531,701,386	G	A
			531,701,395	C	TACCGGGCG
			531,701,415	TACCAGGCA	G
			531,701,429	A	AA
			531,701,436	TG	T
			531,702,306	G	T
	TraesCS3B02G471500	3B	720,177,490	C	G
			720,177,653	A	C
	TraesCS3B02G471900	3B	720,263,094	CGTC	TGTG
	TraesCS3D02G305300	3D	419,464,759	C	–
			419,464,777	C	–
	TraesCS3D02G305400	3D	419,515,860	TTAT	TT
			582,055,996	ATAGT	AT
			582,056,035	C	A
			582,056,048	GTATATAACTAGCG	ATATC
				C	
			582,056,107	C	T
			582,056,130	C	G
			582,056,161	C	T
	TraesCS5A02G424000	5A	609,790,147	GGTCTACGACTGCG	GG
	TraesCS5D02G432600	5D	488,783,912	C	–
			488,784,312	C	–
Serine/threonine protein kinase	TraesCS1A02G080600	1A	63,476,951	CAAAAAACCCT	CAAAAAAAACCCT
	TraesCS1B02G098600	1B	105,108,534	C	A
	TraesCS1D02G082500	1D	64,420,937	ATTTTTTCCA	ATTTTTTTCCA
	TraesCS2B02G124100	2B	92,490,345	A	G
			92,490,898	G	C
	TraesCS2D02G107100	2D	59,450,560	ATG	AG
			59,450,572	A	G
Calcium signaling	TraesCS5D02G269400	5D	372,454,779	GTTTTTTTTTATTTT	–
				CAAA	
Metal ion transport	TraesCS3A02G258400	3A	480,147,175	GTTTTTTTGTTCTA	F
	TraesCS5A02G043000	5A	38,797,696	C	TCAT

Note. DEGs, differentially expressed genes; KRL 3–4, bread wheat germplasm; ROS, reactive oxygen species.

## Conclusion

In summary, our study provided detailed insights into genes, regulatory elements, and various metabolic pathways that could be important for enhanced sodicity stress tolerance in bread wheat genotype KRL3–4. Although many metabolic pathways were altered, lipid metabolism and protein homeostasis-related pathways appear to have a most important role in sodicity tolerance. The genomic information, particularly 19 candidate genes identified in our study, could be further validated using functional genomics approaches for understanding their detailed role in sodicity stress tolerance in wheat.

## Data Availability

The datasets presented in this study can be found in online repositories. The names of the repository/repositories and accession number(s) can be found at: https://www.ncbi.nlm.nih.gov/, PRJNA763033.

## Abbreviations

TF, transcription factor; ROS, Reactive Oxygen species; GO, gene ontology; QC, quality control; BP, biological process; MF, molecular function; CC, cellular component; DEGs, differentially expressed genes; KEGG, Kyoto Encyclopedia of genes and genomes; AS, alternative splicing; SNP, single nucleotide polymorphism; PC-PLA1, phospholipases A1; pPLA2-II, phospholipase A2; KCR, 3-ketoacyl-CoA reductase; KCS, 3-ketoacyl-CoA synthase; GPAT, Glycerol-3-phosphate acyltransferase; DGAT, acyl-CoA:diacylglycerol acyltransferase; SPX, phosphate signaling regulatory protein; PHT1, phosphate transporter 1; NLA, Nitrogen limitation adaptation, GRX, Glutaredoxins; GUX, glucuronosyltransferase; CARE, cis-acting regulatory elements; STPK, Serine-threonine protein kinases; TAG, Triacylglycerol; BBI, Bowman-Birk protease inhibitor; AGPs, Arabinogalactan proteins; DRE, dehydration-responsive element.
